# The genome sequence of the ichneumon wasp *Buathra laborator *(Thunberg, 1822)

**DOI:** 10.12688/wellcomeopenres.18674.1

**Published:** 2023-01-06

**Authors:** Gavin R. Broad, Natural History Museum Genome Acquisition Lab, Darwin Tree of Life Barcoding collective, Wellcome Sanger Institute Tree of Life programme, Wellcome Sanger Institute Scientific Operations: DNA Pipelines collective, Tree of Life Core Informatics collective, Darwin Tree of Life Consortium

**Affiliations:** 1 Natural History Museum, London, UK

**Keywords:** Buathra laborator, wasp, genome sequence, chromosomal, Hymenoptera

## Abstract

We present a genome assembly from an individual *Buathra laborator* (Arthropoda; Insecta; Hymenoptera; Ichneumonidae). The genome sequence is 330 megabases in span. Over 60% of the assembly is scaffolded into 11 chromosomal pseudomolecules. The mitochondrial genome has also been assembled and is 35.8 kilobases in length.

## Species taxonomy

Eukaryota; Metazoa; Ecdysozoa; Arthropoda; Hexapoda; Insecta; Pterygota; Neoptera; Endopterygota; Hymenoptera; Apocrita; Parasitoida; Ichneumonoidea; Ichneumonidae; Cryptinae; Cryptina; *Buathra*; *Buathra laborator* Thunberg, 1822) (NCBI:txid1419289).

## Background

*In Britain, Buathra laborator* is a common and widespread Darwin wasp species. Although there is no organised recording of most ichneumonid wasps, data are available in museum collections (e.g., Schwarz & Shaw, 1998) to demonstrate its wide distribution and relatively long flight period, from April to August. This is one of our largest species of the ichneumonid subfamily Cryptinae, particularly conspicuous because it is found in rather open areas and often on flowers. Both sexes are basically black with red legs, females have a rather long ovipositor, males have bright white markings on the face and on the hind tarsus. They can be confused with some species of *Cryptus* (which are usually smaller) and with the very similar *Buathra tarsoleucos* (Schrank); separation from *B. tarsoleucos* depends on close examination of the female ovipositor tip or, in males, details of the propodeum ( Schwarz, 1990). In Britain, *B. tarsoleucos* seems to be rare ( Schwarz & Shaw, 1998).

Despite its conspicuousness, *B. laborator* has never been reared in Europe. There is an American rearing from the geometrid Western Carpet moth *Melanolophia imitate* (Walker) ( Townes & Townes, 1962), which pupates in leaf litter. Available host records in the literature (summarised by Yu *et al.*, 2016) seem to relate to misidentifications. *Buathra tarsoleucos* is a parasitoid of the pupae of sand wasps (Hymenoptera: Sphecidae) ( Casiraghi *et al.*, 2001). As with B *. tarsoleucos* and species of the closely related ( Santos, 2017) genus *Cryptus*, the hosts of *B. laborator* will undoubtedly be cocooned pupae hidden in the soil; most Cryptinae are idiobiont ectoparasitoids, i.e., the host is permanently paralysed and the wasp larva feeds externally (summarised by ( Broad *et al.*, 2018)).

Outside of Britain, *B. laborator* has a very wide range across Europe, temperate Asia and North America (summarised by ( Yu *et al.*, 2016)). Several genes have previously been sequenced, placing *B. laborator* within a phylogenetic framework for Cryptinae ( Santos, 2017) and Ichneumonidae ( Quicke *et al.*, 2009), but this is only the second complete genome for an ichneumonid, or Darwin wasp (see ( Klopfstein *et al.*, 2019)); both *B. laborator* and *Ichneumon xanthorius* Forster ( Broad, 2022) are classified within the informal ichneumoniformes grouping of subfamilies (e.g., Quicke *et al.*, 2009).

The genome of the wasp *B. laborator* was sequenced as part of the Darwin Tree of Life Project, a collaborative effort to sequence all named eukaryotic species in the Atlantic Archipelago of Britain and Ireland.

## Genome sequence report

The genome was sequenced from a female *B. laborator* collected from Hartslock, UK (latitude 51.51, longitude –1.11) ( Wellcome Sanger Institute, 2022). A total of 49-fold coverage in Pacific Biosciences single-molecule HiFi long reads and 119-fold coverage in 10X Genomics read clouds were generated. Primary assembly contigs were scaffolded with chromosome conformation Hi-C data. Manual assembly curation corrected 23 missing/misjoins, reducing the scaffold number by 14.84% and increasing the scaffold N50 by 33.3%.

The final assembly has a total length of 330 Mb in 109 sequence scaffolds with a scaffold N50 of 17 Mb ( Table 1). Most (68.92%) of the assembly sequence was assigned to 11 chromosomal-level scaffolds ( Figure 2– Figure 5; Table 2). Chromosome-scale scaffolds confirmed by the Hi-C data are named in order of size. Several repeat types can be seen to cluster by Hi-C yet have no association with the defined chromosomes. These repeats make up a large proportion of the assembly ( Figure 5). While not fully phased, the assembly deposited is of one haplotype. Contigs corresponding to the second haplotype have also been deposited. The assembly has a BUSCO v5.3.2 ( Manni *et al.*, 2021) completeness of 95.9% (single 95.7%, duplicated 0.3%) using the hymenoptera_odb10 reference set.

**Table 1.** Genome data for *B. laborator*, iyBuaLabo1.1.

Project accession data	
Assembly identifier	iyBuaLabo1.1
Species	*Buathra laborator*
Specimen	iyBuaLabo1
NCBI taxonomy ID	1419289
BioProject	PRJEB50481
BioSample ID	SAMEA8534297
Isolate information	Thorax (DNA sequencing), Head (Hi-C)
Raw data accessions	
PacificBiosciences SEQUEL II	ERR8482057
10X Genomics Illumina	ERR8373780–ERR8373783
Hi-C Illumina	ERR8373784
Genome assembly	
Assembly accession	GCA_934046635.1
*Accession of alternate haplotype*	GCA_934046145.1
Span (Mb)	330
Number of contigs	141
Contig N50 length (Mb)	10.4
Number of scaffolds	109
Scaffold N50 length (Mb)	17.0
Longest scaffold (Mb)	33.2
BUSCO *	C:95.9%[S:95.7%,D:0.3%],F:1.2 %,M:2.8%,n:5,991

* BUSCO scores based on the hymenoptera_odb10 BUSCO set using v5.3.2. C = complete [S = single copy, D = duplicated], F = fragmented, M = missing, n = number of orthologues in comparison. A full set of BUSCO scores is available at https://blobtoolkit.genomehubs.org/view/iyBuaLabo1.1/dataset/CAKOHB01/busco.

**Table 2.** Chromosomal pseudomolecules in the genome assembly of *B. laborator*, iyBuaLabo1.

INSDC accession	Chromosome	Size (Mb)	GC%
OW203892.1	1	33.19	43.8
OW203893.1	2	25.36	41.3
OW203894.1	3	24.72	40.9
OW203895.1	4	20.43	40.4
OW203896.1	5	22.1	39.9
OW203897.1	6	18.7	41.3
OW203898.1	7	17.92	40.5
OW203899.1	8	17.03	40.9
OW203900.1	9	16.77	40.4
OW203901.1	10	16.25	40.4
OW203902.1	11	13.03	40.6
OW203903.1	MT	0.04	16.7
-	unplaced	104.41	45.9

**Figure 1.**
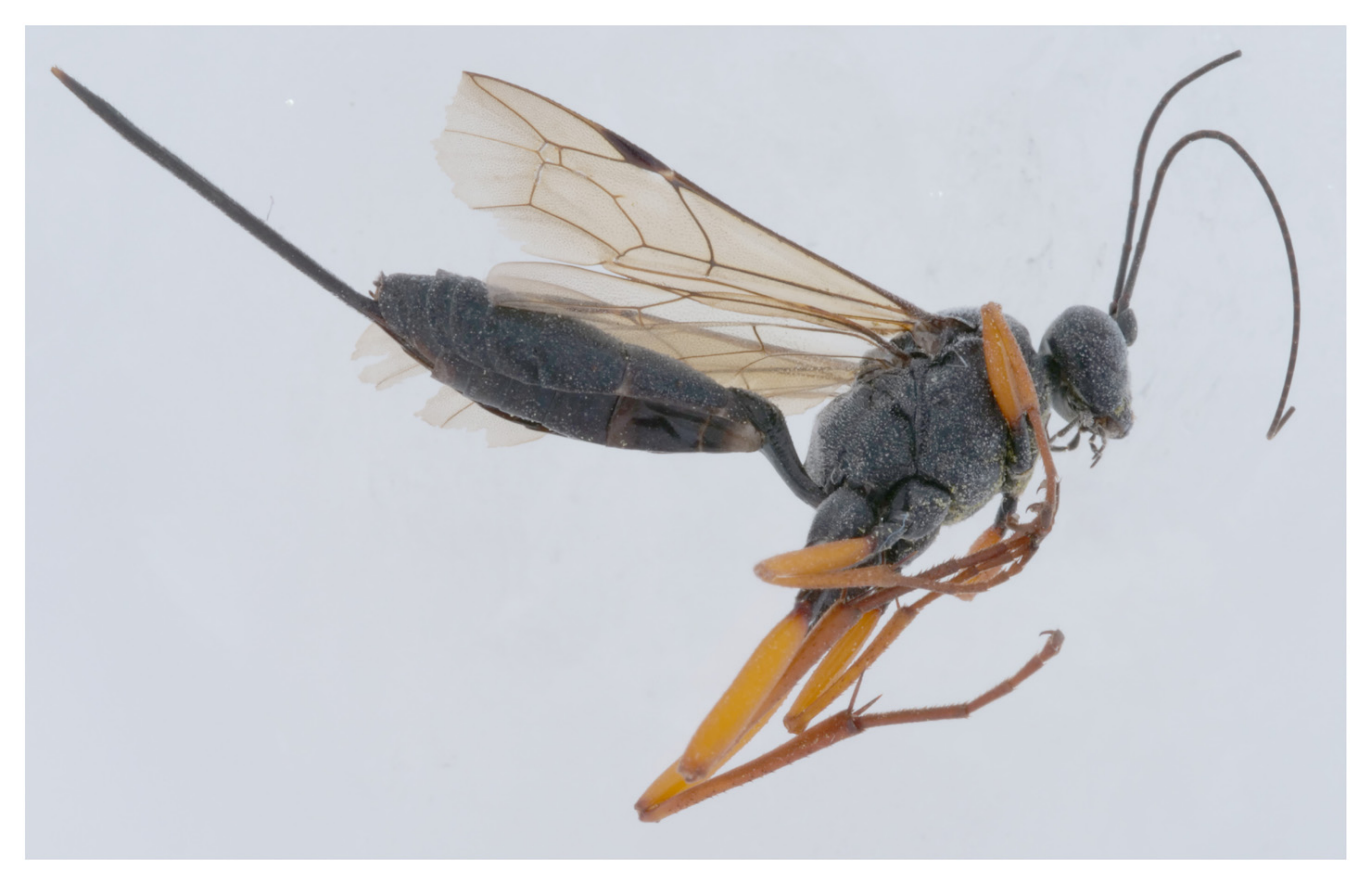
Image of the *B. laborator*, iyBuaLabo1 specimen used for genome sequencing.

**Figure 2.**
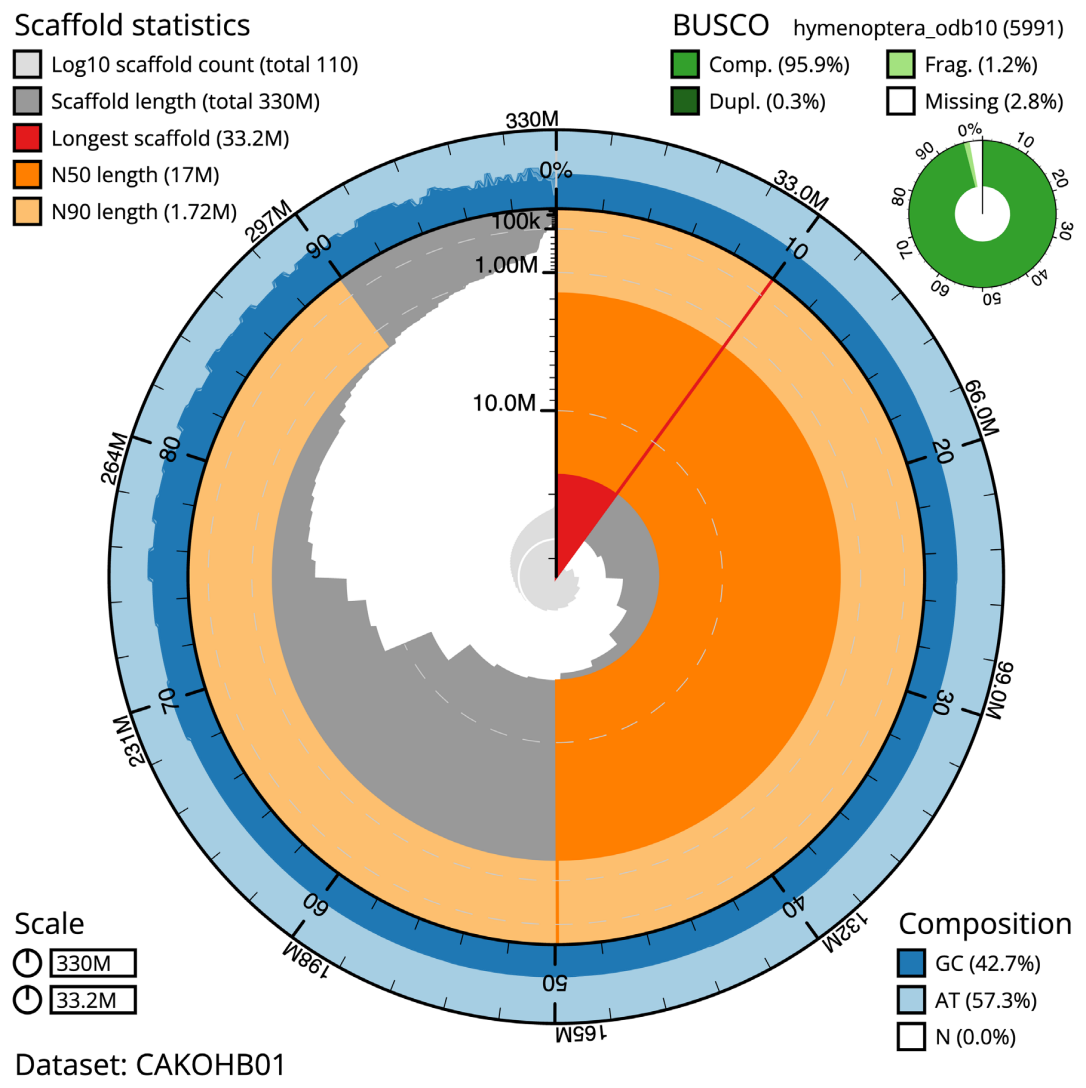
Genome assembly of *B. laborator*, iyBuaLabo1.1: metrics. The BlobToolKit Snailplot shows N50 metrics and BUSCO gene completeness. The main plot is divided into 1,000 size-ordered bins around the circumference with each bin representing 0.1% of the 329,931,003 bp assembly. The distribution of chromosome lengths is shown in dark grey with the plot radius scaled to the longest chromosome present in the assembly (33,193,724 bp, shown in red). Orange and pale-orange arcs show the N50 and N90 chromosome lengths (17,025,623 and 1,720,325 bp), respectively. The pale grey spiral shows the cumulative chromosome count on a log scale with white scale lines showing successive orders of magnitude. The blue and pale-blue area around the outside of the plot shows the distribution of GC, AT and N percentages in the same bins as the inner plot. A summary of complete, fragmented, duplicated and missing BUSCO genes in the hymenoptera_odb10 set is shown in the top right. An interactive version of this figure is available at https://blobtoolkit.genomehubs.org/view/iyBuaLabo1.1/dataset/CAKOHB01/snail.

**Figure 3.**
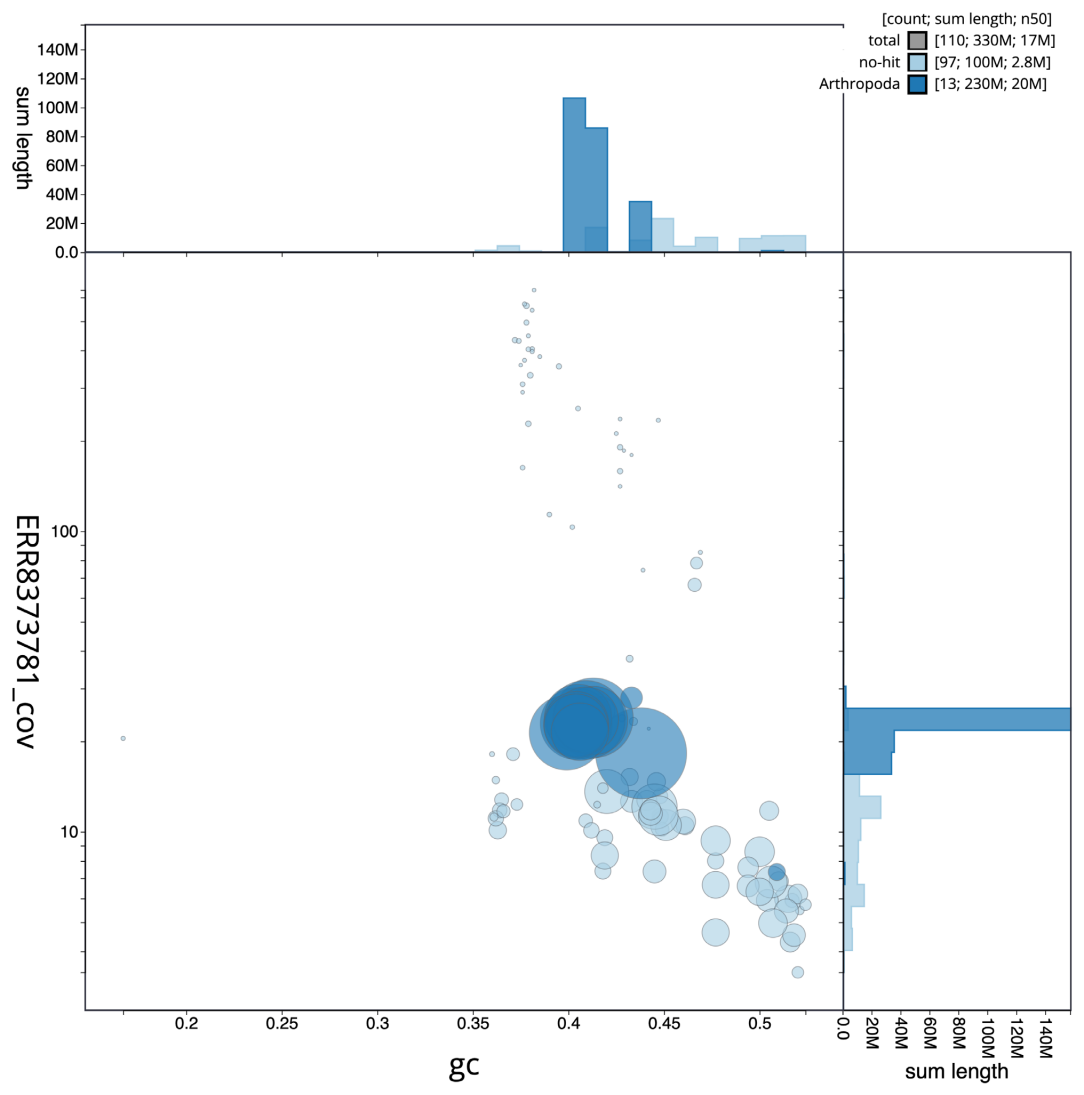
Genome assembly of *B. laborator*, iyBuaLabo1.1: GC coverage. BlobToolKit GC-coverage plot. An interactive version of this figure is available at https://blobtoolkit.genomehubs.org/view/iyBuaLabo1.1/dataset/CAKOHB01/blob.

**Figure 4.**
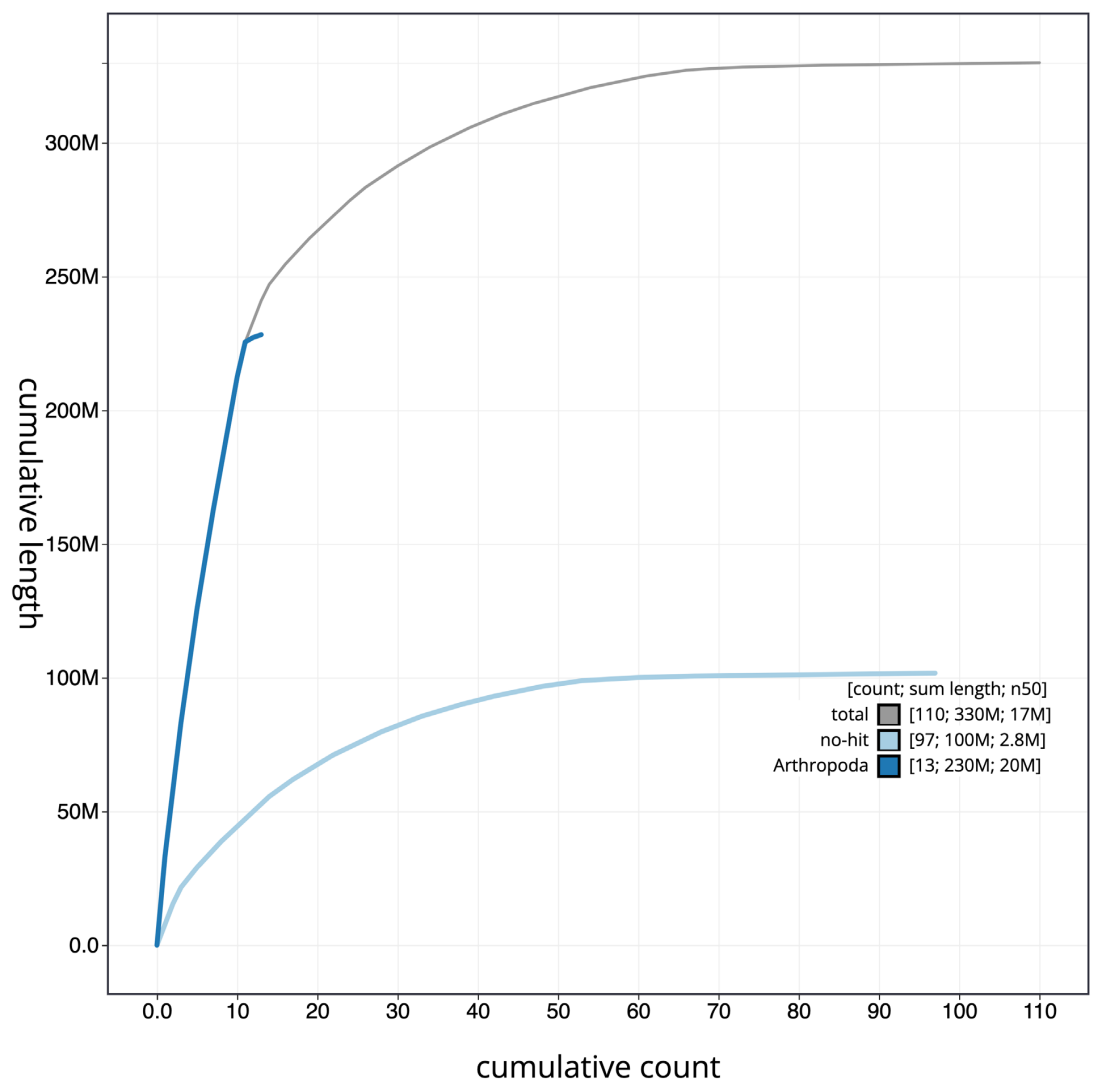
Genome assembly of *B. laborator*, iyBuaLabo1.1: cumulative sequence. BlobToolKit cumulative sequence plot. An interactive version of this figure is available at https://blobtoolkit.genomehubs.org/view/iyBuaLabo1.1/dataset/CAKOHB01/cumulative.

**Figure 5.**
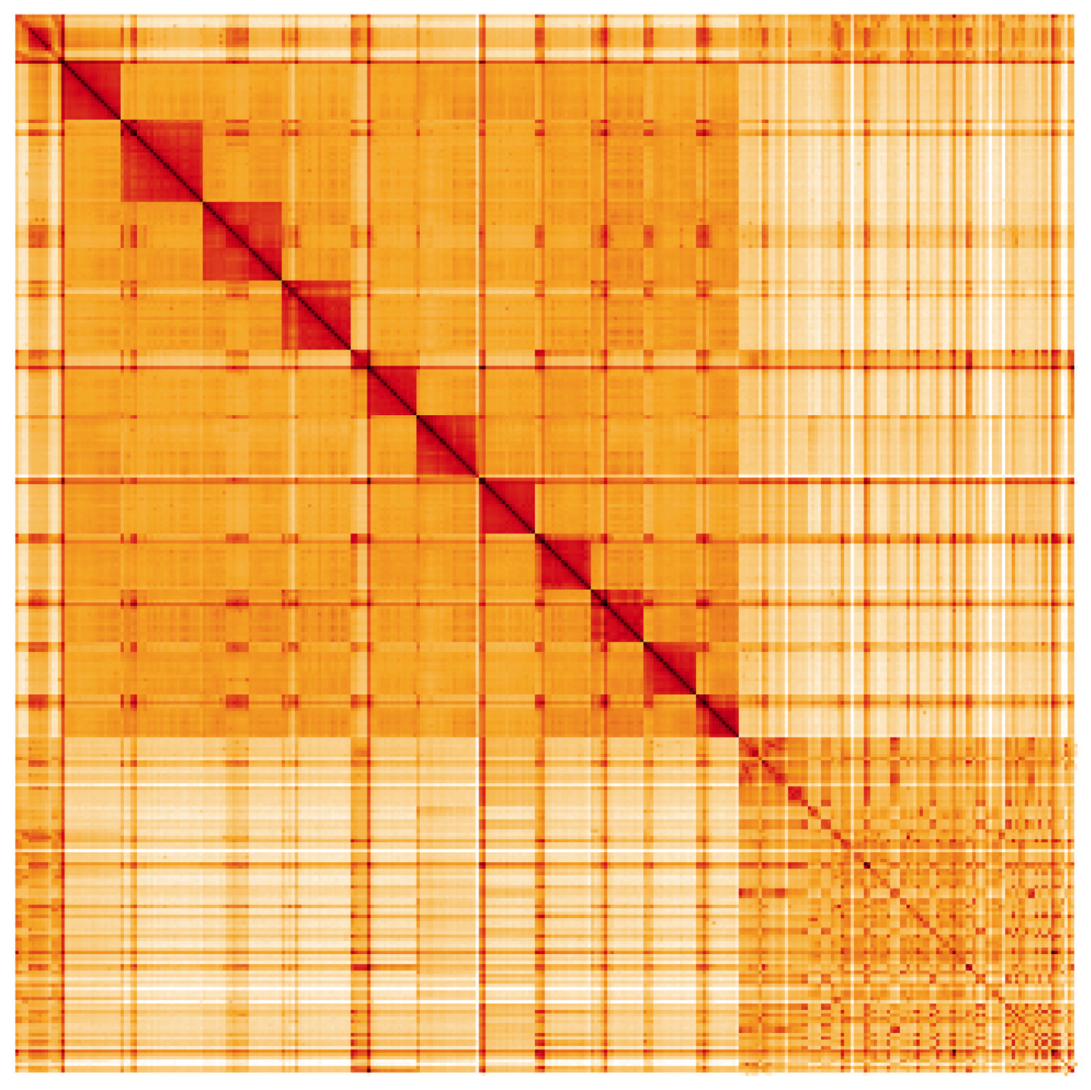
Genome assembly of *B. laborator*, iyBuaLabo1.1: Hi-C contact map. Hi-C contact map of the iyBuaLabo1.1 assembly, visualised using HiGlass. Chromosomes are given in order of size from left to right and top to bottom. An interactive version of this plot can be accessed at https://genome-note-higlass.tol.sanger.ac.uk/l/?d=ZLhYoonSSDebWPGX2ItCHQ.

## Methods

### Sample acquisition and nucleic acid extraction

An individual *B. laborator* (iyBuaLabo1) ( Figure 1) was collected and identified by Matt Smith from Hartslock, UK (latitude 51.51, longitude –1.11). The sample was caught using an aerial net and preserved in liquid nitrogen.

DNA was extracted at the Tree of Life laboratory, Wellcome Sanger Institute. The iyBuaLabo1 sample was weighed and dissected on dry ice with head tissue set aside for Hi-C sequencing. The thorax tissue was disrupted using a Nippi Powermasher fitted with a BioMasher pestle. High molecular weight (HMW) DNA was extracted using the Qiagen MagAttract HMW DNA extraction kit. Low molecular weight DNA was removed from a 20 ng aliquot of extracted DNA using 0.8X AMpure XP purification kit prior to 10X Chromium sequencing; a minimum of 50 ng DNA was submitted for 10X sequencing. HMW DNA was sheared into an average fragment size of 12–20 kb in a Megaruptor 3 system with speed setting 30. Sheared DNA was purified by solid-phase reversible immobilisation using AMPure PB beads with a 1.8X ratio of beads to sample to remove the shorter fragments and concentrate the DNA sample. The concentration of the sheared and purified DNA was assessed using a Nanodrop spectrophotometer and Qubit Fluorometer and Qubit dsDNA High Sensitivity Assay kit. Fragment size distribution was evaluated by running the sample on the FemtoPulse system.

### Sequencing

Pacific Biosciences HiFi circular consensus and 10X Genomics read cloud DNA sequencing libraries were constructed according to the manufacturers’ instructions. DNA sequencing was performed by the Scientific Operations core at the WSI on Pacific Biosciences SEQUEL II (HiFi) and Illumina NovaSeq 6000 (10X) instruments. Hi-C data were also generated from head tissue of iyBuaLabo1 using the Arimav2 kit and sequenced on the Illumina NovaSeq 6000 instrument.

### Genome assembly

Assembly was carried out with Hifiasm ( Cheng *et al.*, 2021). Haplotypic duplication was identified and removed with purge_dups ( Guan *et al.*, 2020). Scaffolding with Hi-C data ( Rao *et al.*, 2014) was carried out with SALSA2 ( Ghurye *et al.*, 2019). The Hi-C scaffolded assembly was polished with the 10X Genomics Illumina data by aligning to the assembly with longranger align, calling variants with freebayes ( Garrison & Marth, 2012). One round of Illumina polishing was applied. The mitochondrial genome was assembled with MitoHiFi ( Uliano-Silva *et al.*, 2021), which performed annotation using MitoFinder ( Allio *et al.*, 2020). The assembly was checked for contamination as described previously ( Howe *et al.*, 2021). Manual curation ( Howe *et al.*, 2021) was performed using HiGlass ( Kerpedjiev *et al.*, 2018) and Pretext. The genome was analysed within the BlobToolKit environment ( Challis *et al.*, 2020). Table 3 contains a list of all software tool versions used, where appropriate.

**Table 3.** Software tools and versions used.

Software tool	Version	Source
BlobToolKit	3.2.6	Challis *et al.*, 2020
freebayes	1.3.1-17-gaa2ace8	Garrison and Marth, 2012
HiCanu	0.15.3	Nurk *et al.*, 2020
HiGlass	1.11.6	Kerpedjiev *et al.*, 2018
Long Ranger ALIGN	2.2.2	https://support.10xgenomics.com/genome-exome/software/pipelines/latest/advanced/other-pipelines
MitoHiFi	1.0	Uliano-Silva *et al.*, 2021
PretextView	0.2.x	Harry, 2022
purge_dups	1.2.3	Guan *et al.*, 2020
SALSA2	2.2	Ghurye *et al.*, 2019

### Ethics/compliance issues

The materials that have contributed to this genome note have been supplied by a Darwin Tree of Life Partner. The submission of materials by a Darwin Tree of Life Partner is subject to the Darwin Tree of Life Project Sampling Code of Practice. By agreeing with and signing up to the Sampling Code of Practice, the Darwin Tree of Life Partner agrees they will meet the legal and ethical requirements and standards set out within this document in respect of all samples acquired for, and supplied to, the Darwin Tree of Life Project. Each transfer of samples is further undertaken according to a Research Collaboration Agreement or Material Transfer Agreement entered into by the Darwin Tree of Life Partner, Genome Research Limited (operating as the Wellcome Sanger Institute), and in some circumstances other Darwin Tree of Life collaborators.

## Data Availability

European Nucleotide Archive: *Buathra laborator*. Accession number PRJEB50481; https://identifiers.org/ena.embl/PRJEB50481. ( Wellcome Sanger Institute, 2022). The genome sequence is released openly for reuse. The *Buathra laborator* genome sequencing initiative is part of the Darwin Tree of Life (DToL) project. All raw sequence data and the assembly have been deposited in INSDC databases. The genome will be annotated using available RNA-Seq data and presented through the Ensembl pipeline at the European Bioinformatics Institute. Raw data and assembly accession identifiers are reported in Table 1. Members of the Natural History Museum Genome Acquisition Lab are listed here: https://doi.org/10.5281/zenodo.4790042. Members of the Darwin Tree of Life Barcoding collective are listed here: https://doi.org/10.5281/zenodo.4893703. Members of the Wellcome Sanger Institute Tree of Life programme are listed here: https://doi.org/10.5281/zenodo.4783585. Members of Wellcome Sanger Institute Scientific Operations: DNA Pipelines collective are listed here: https://doi.org/10.5281/zenodo.4790455. Members of the Tree of Life Core Informatics collective are listed here: https://doi.org/10.5281/zenodo.5013541. Members of the Darwin Tree of Life Consortium are listed here: https://doi.org/10.5281/zenodo.4783558.

## References

[ref-1] Allio R Schomaker-Bastos A Romiguier J : MitoFinder: Efficient automated large-scale extraction of mitogenomic data in target enrichment phylogenomics. *Mol Ecol Resour.* 2020;20(4):892–905. 10.1111/1755-0998.13160 32243090 PMC7497042

[ref-2] Broad G Darwin Tree of Life Barcoding collective : The genome sequence of a parasitoid wasp, *Ichneumon xanthorius* Forster, 1771 [version 1; peer review: 3 approved]. *Wellcome Open Res.* 2022;7:47. 10.12688/wellcomeopenres.17683.1 35419493 PMC8984213

[ref-3] Broad GR Shaw MR Fitton MG : The ichneumonid wasps of Britain and Ireland (Hymenoptera: Ichneumonidae): Their classification and biology.Telford: Royal Entomological Society and Field Studies Council.2018.

[ref-4] Casiraghi M Andrietti F Bonasoro F : A note on host detection by *Buathra tarsoleuca* (Schrank) (Hymenoptera: Ichneumonidae), a parasite of *Ammophila sabulosa* (L.) and *Podalonia affinis* (Kirby) (Hymenoptera: Sphecidae). *J Insect Behav.* 2001;14(3):299–312.

[ref-5] Challis R Richards E Rajan J : BlobToolKit - Interactive Quality Assessment of Genome Assemblies. *G3 (Bethesda).* 2020;10(4):1361–1374. 10.1534/g3.119.400908 32071071 PMC7144090

[ref-6] Cheng H Concepcion GT Feng X : Haplotype-resolved *de novo* assembly using phased assembly graphs with hifiasm. *Nat Methods.* 2021;18(2):170–175. 10.1038/s41592-020-01056-5 33526886 PMC7961889

[ref-7] Garrison E Marth G : Haplotype-based variant detection from short-read sequencing. 2012. 10.48550/arXiv.1207.3907

[ref-8] Ghurye J Rhie A Walenz BP : Integrating Hi-C links with assembly graphs for chromosome-scale assembly. *PLoS Comput Biol.* 2019;15(8):e1007273. 10.1371/journal.pcbi.1007273 31433799 PMC6719893

[ref-9] Guan D McCarthy SA Wood J : Identifying and removing haplotypic duplication in primary genome assemblies. *Bioinformatics.* 2020;36(9):2896–2898. 10.1093/bioinformatics/btaa025 31971576 PMC7203741

[ref-10] Harry E : PretextView (Paired REad TEXTure Viewer): A desktop application for viewing pretext contact maps. 2022; Accessed: 19 October 2022. Reference Source

[ref-11] Howe K Chow W Collins J : Significantly improving the quality of genome assemblies through curation. *GigaScience.* Oxford University Press,2021;10(1):giaa153. 10.1093/gigascience/giaa153 33420778 PMC7794651

[ref-12] Kerpedjiev P Abdennur N Lekschas F : HiGlass: web-based visual exploration and analysis of genome interaction maps. *Genome Biol.* 2018;19(1):125. 10.1186/s13059-018-1486-1 30143029 PMC6109259

[ref-13] Klopfstein S Santos BF Shaw MR : Darwin wasps: a new name heralds renewed efforts to unravel the evolutionary history of Ichneumonidae. *Entomol Commun.* 2019;1:ec01006. 10.37486/2675-1305.ec01006

[ref-14] Manni M Berkeley MR Seppey M : BUSCO Update: Novel and Streamlined Workflows along with Broader and Deeper Phylogenetic Coverage for Scoring of Eukaryotic, Prokaryotic, and Viral Genomes. * Mol Biol Evol.* 2021;38(10):4647–4654. 10.1093/molbev/msab199 34320186 PMC8476166

[ref-15] Nurk S Walenz BP Rhie A : HiCanu: Accurate assembly of segmental duplications, satellites, and allelic variants from high-fidelity long reads. *Genome Res.* 2020;30(9):1291–1305. 10.1101/gr.263566.120 32801147 PMC7545148

[ref-16] Quicke DL Laurenne NM Fitton MG : A thousand and one wasps: a 28S rDNA and morphological phylogeny of the Ichneumonidae (Insecta: Hymenoptera) with an investigation into alignment parameter space and elision. *J Nat Hist.* 2009;43(23–24):1305–1421. 10.1080/00222930902807783

[ref-17] Rao SS Huntley MH Durand NC : A 3D map of the human genome at kilobase resolution reveals principles of chromatin looping. *Cell.* 2014;159(7):1665–1680. 10.1016/j.cell.2014.11.021 25497547 PMC5635824

[ref-18] Santos BF : Phylogeny and reclassification of Cryptini (Hymenoptera, Ichneumonidae, Cryptinae), with implications for ichneumonid higher-level classification. *Syst Entomol.* 2017;42(4):650–676. 10.1111/syen.12238

[ref-19] Schwarz M : Bemerkungen zur Systematik und Taxonomie westpaläarktischer Schlupfwespen (Ichneumonidae, Hymenoptera). *Linzer Biologische Beiträge.* 1990;22:59–67.

[ref-20] Schwarz M Shaw MR : Western Palaearctic Cryptinae (Hymenoptera: Ichneumonidae) in the National Museums of Scotland, with nomenclatural changes, taxonomic notes, rearing records and special reference to the British check list. Part 1. Tribe Cryptini. *Entomol's Gaz.* 1998;49:101–127. Reference Source

[ref-21] Townes HK Townes M : Ichneumon-flies of America north of Mexico: 3. Subfamily Gelinae, tribe Mesostenini. *United States National Museum Bulletin.* 1962;216(3):1–602. 10.5479/si.03629236.216.1-3

[ref-22] Uliano-Silva M : MitoHiFi. 2021. Accessed: 19 October 2022. Reference Source

[ref-23] Wellcome Sanger Institute: The genome sequence of the ichneumon wasp *Buathra laborator*, (Thunberg, 1822). *European Nucleotide Archive.* [dataset].2022, accession number PRJEB50481. Reference Source

[ref-24] Yu DS van Achterberg C Horstmann K : Taxapad 2016, Ichneumonoidea 2015. Database on flash-drive.Ontario, Canada: Nepean.2016; Accessed: 7 November 2022.

