# Supplementary material for: The genome sequence of the ichneumon wasp Buathra laborator (Thunberg, 1822)
Source: Wellcome Open Res. 2023 Jan 6;8:7. [Version 1] doi: 10.12688/wellcomeopenres.18674.1 (PMC10206444; doi:10.12688/wellcomeopenres.18674.1)
Supplement: Peer review history

# Peer review: Reviewer response for version 1

**DOI:** 10.21956/wellcomeopenres.20709.r57942

**Published:** 2023-05-23

**Authors:** Xinhai Ye

**Affiliations:** 1 Zhejiang University, Hangzhou, Zhejiang, China

This short note reported a high-quality genome assembly of the parasitoid wasp *Buathra laborator*. And this genome project is part of the Darwin Tree of Life Project. This paper presented the morphological characteristics and genome information of this wasp, and this genome data will undoubtedly advance the genome research of Hymenoptera. Here are just a few comments.

1. In the introduction, the morphological characteristics of the parasitic wasp are described in detail, but the host information of the wasp is not explained. I think this information needs to be added.
2. Only 69% of the assembled genome was assigned to 11 chromosomal level scaffolds, which was lower than I expected. So I suggest more discussion or explanation.
3. The sex of the sequenced sample needs to be reported.

Are sufficient details of methods and materials provided to allow replication by others?

Yes

Is the rationale for creating the dataset(s) clearly described?

Yes

Are the datasets clearly presented in a useable and accessible format?

Yes

Are the protocols appropriate and is the work technically sound?

Yes

Reviewer Expertise:

Parasitoid wasp genomics, Genome sequencing

I confirm that I have read this submission and believe that I have an appropriate level of expertise to confirm that it is of an acceptable scientific standard.

# Peer review: Reviewer response for version 1

**DOI:** 10.21956/wellcomeopenres.20709.r54132

**Published:** 2023-01-31

**Authors:** Gaelen R Burke

**Affiliations:** 1 Department of Entomology, University of Georgia, Athens, GA, USA

The genome assembly report focuses upon *Buathra laborator*, an ichneumonid wasp belonging to the Cryptinae. Below are some minor comments for improving the publication:

 “There is an American rearing from the geometrid Western Carpet moth *Melanolophia imitate* (Walker)”

 This sentence sounds a little strange. I suggest saying that this species has been reared in North America in the past (rather than mentioning the “American”).

 “Available host records in the literature (summarised by Yu *et al.*, 2016) seem to relate to misidentifications”

 This sentence could be more clear. In what way do the host records related to misidentifications?

 “but this is only the second complete genome for an ichneumonid, or Darwin wasp“

 I don’t think this is true. This may be the second ichneumonid genome that has been assembled at a chromosomal level, but several have been sequenced to a level that would be considered complete by community standards (just more fragmented).

 I suggest slight re-organization of the background to have paragraphs focus upon themes a little better. There is information about distribution and habitat in two different paragraphs, paired with phylogenetic placement in the later paragraph. Other sentences joined by a semicolon seem unrelated in content at the beginning and end.

 The genome sequence itself is assembled appropriately and the figures and tables describing the assembly are useful. The assembly statistics show that the genome is in a high quality state and will be a good community resource. The places that scientists can access the data are clearly indicated.

 Overall this genome assembly report is of good quality and will be a useful addition to the community, improving the availability of genomes for Darwin’s wasps.

Are sufficient details of methods and materials provided to allow replication by others?

Yes

Is the rationale for creating the dataset(s) clearly described?

Yes

Are the datasets clearly presented in a useable and accessible format?

Yes

Are the protocols appropriate and is the work technically sound?

Yes

Reviewer Expertise:

Insect genomics and host-microbe interactions

I confirm that I have read this submission and believe that I have an appropriate level of expertise to confirm that it is of an acceptable scientific standard.
